# Muscle synergy differences between voluntary and reactive backward stepping

**DOI:** 10.1038/s41598-021-94699-z

**Published:** 2021-07-29

**Authors:** Shuaijie Wang, Gonzalo Varas-Diaz, Tanvi Bhatt

**Affiliations:** Department of Physical Therapy, University of Illinois at Chicago, 1919 W Taylor St (M/C 898), Chicago, IL 60612 USA

**Keywords:** Motor control, Electromyography - EMG

## Abstract

Reactive stepping responses are essential to prevent falls after a loss of balance. It has previously been well described that both voluntary and reactive step training could improve the efficacy of reactive stepping in different populations. However, the effect of aging on neuromuscular control during voluntary and reactive stepping remains unclear. Electromyography (EMG) signals during both backward voluntary stepping in response to an auditory cue and backward reactive stepping elicited by a forward slip-like treadmill perturbation during stance were recorded in ten healthy young adults and ten healthy older adults. Using muscle synergy analysis, we extracted the muscle synergies for both voluntary and reactive stepping. Our results showed that fewer muscle synergies were used during reactive stepping than during voluntary stepping in both young and older adults. Minor differences in the synergy structure were observed for both voluntary and reactive stepping between age groups. Our results indicate that there is a low similarity of muscle synergies between voluntary stepping and reactive stepping and that aging had a limited effect on the structure of muscle synergies. This study enhances our understanding of the neuromuscular basis of both voluntary and reactive stepping as well as the potential effect of aging on neuromuscular control during balance tasks.

## Introduction

Unexpected perturbations during gait, such as trips and slips, are the main cause of falls in heathy older adults^1^. Slips account for 40% of falls in community-dwelling older adults, with this incidence becoming increasingly prevalent as individuals age^1,2^. Similarly, for the older adults residing in long-term care, external perturbations also account for ~ 30% of falls^3^. Reactive balance control in response to a perturbation requires significant kinematic and neuromuscular changes to regain stability^4,5^, and an adequate stepping response has previously been viewed as a critical factor in preventing falls^6^. Besides reactive stepping, appropriately timed and directed steps could be initiated voluntarily to avoid a fall^7,8^. In order to improve reactive balance control and voluntary stepping, several kinds of reactive and voluntary stepping training protocols have been developed for older adults^9–14^.

Previous studies have found that both reactive and voluntary step training could improve balance and reactive responses upon experiencing an unexpected perturbation^14–18^, leading to a reduction of falls among older adults by approximately 50%^19^. Both voluntary and reactive step training has shown to be effective for fall prevention in populations at high risk of falls. However, few studies have reported differences in the sensorimotor strategies used in voluntary stepping compared to reactive stepping responses^20^. One of the major differences between these stepping strategies is the role of anticipatory postural adjustments (APA), defined as anticipatory activation and inhibition of postural muscles prior to self-triggered or self-inflicted posture perturbations. It was reported that APA could precede the initiation of voluntary stepping in both amplitude and duration according to the distance and speed of the stepping action^21,22^. On the other hand, it has been well described that externally-induced reactive stepping responses have a shorter initiation time than voluntary stepping^23^ and the APAs are significantly less effective during reactive stepping responses^24,25^, thus indicating that the motor control strategies might be different between these two types of stepping. Additionally, contrary to voluntary movements which are initiated and organized by cortical sensorimotor regions^26^, studies have shown that the earliest part of the postural response after an external perturbation likely arises from the spinal and subcortical regions instead of the cortex. A direct cortical loop might not be involved in the initial phase of postural response to external perturbations but only involved in the later phases of the responses^27–29^. These differences in contributions from APAs and neurophysiological correlates between voluntary and reactive movement might further indicate differences in the complexity of movement control between them. Moreover, evidence indicates that the movement complexity (assessed using sample entropy) decreased along with environmental modifications (walking over inclined surfaces)^30^. Additionally, it has been well described that reactive movement control differs from voluntary movement in that it is inherently a feedback sensorimotor process where muscle activity is activated in direct response to task-level error in less time than voluntary movements^31,32^. Thus, it could be postulated that the reactive stepping response might require a reduced complexity in motor control compared to voluntary stepping, especially responses induced by larger perturbations (higher velocity/acceleration). Altogether, it is reasonable to postulate that the motor strategies used in voluntary stepping and reactive stepping might differ, at least partially. A comparison of these two stepping responses by examination of the muscle synergies during the motor response could verify such postulation.

Muscle synergies represent the lowest level of the motor control hierarchy and may provide a mechanism by which task-level goals are translated into complex execution-level patterns of muscle activity^33^. Several studies have demonstrated that muscle synergies, defined as low-dimensional modules formed by muscles synchronously being activated^34,35^, could be used by the nervous system to generate motor output patterns during postural tasks. In this context, it has been reported that changes in the activation of individual muscles represent variations in the amplitude of descending neural commands that activate individual muscle synergies. Additionally, it was found that the composition and temporal activation of the muscles synergies during postural tasks were highly consistent across individuals, suggesting that muscle synergies represent a general neural strategy underlying muscle coordination during postural tasks^33^. Although muscle synergies have been examined separately for voluntary stepping (e.g., self-paced or fast-paced with an auditory cue) and recovery stepping^36–38^, the perturbation intensity used in these studies was not sufficient to trigger a recovery step^39^. It remains unclear what kind of muscle synergies were recruited by voluntary step and recovery step during large-magnitude perturbations. As cluster analysis could identify the representative synergy vectors for different condition^40^, hence, the clustered muscle synergies (or the repertory of muscle synergies) will be compared between voluntary stepping and recovery stepping to investigate whether the same repertory of muscle synergies are presented in both stepping.

Further, it has not yet been described whether aging affects the patterns of muscle synergies during voluntary and reactive stepping. Age-related deteriorations in volitional and reactive balance control and gait are well-documented^41–45^. Previous studies have found that age-induced deterioration in postural control and movement control (e.g., body sway, gait, and reach movements) were related to altered activation of the involved antagonistic muscles and changes in the muscle co-activation patterns^46–48^. Other studies also have found differences in muscle activation patterns between young and old for stepping tasks^49,50^. Further, a few studies have reported the difference in muscle synergies between individuals with and without motor function impairment^51–53^ by comparing their repertory of muscle synergies. Considering the age-related deterioration in motor functions, it is possible that there might be differences in the repertory of muscle synergies between young and older adults for voluntary and reactive stepping.

The purposes of this study were to investigate the differences in neuromuscular control between voluntary and reactive backward stepping, as well as to investigate the effect of aging on the neuromuscular control of both stepping responses. First, we hypothesized that there would be differences in neuromuscular control between reactive stepping and voluntary stepping, indicated by differences in structure and number of clustered muscle synergies for each condition. Additionally, we further hypothesized that there would be a change in neuromuscular control of the stepping strategies due to aging, specifically, the repertory of muscle synergies in older adults would change compared to young adults during their reactive and voluntary backward stepping.

## Experimental procedures

### Subjects

Fifteen healthy young adults and fifteen ambulatory, community-dwelling older adults participated in this study and were all right-leg dominant (determined by self-report of the preferred leg for kicking a ball). They were screened via a questionnaire before the experiment to exclude individuals with any neurological, musculoskeletal, cardiopulmonary, or other systemic disorders. Participants whose EMG signals contained missing channels (n = 6) or included artifacts (n = 4) were also excluded. Ten young and ten older participants were included in this study, and demographics for these participants are presented in Table 1. The study was carried out in accordance with the Declaration of Helsinki of 1975, and all participants were provided written informed consent. Both the experimental protocols and the informed consent were approved by the Institutional Review Board of the University of Illinois at Chicago.

**Table 1:** Comparisons of the demographics in means ± SD between young and older adults. Independent *t* test was used.

	Young	Older	p value
Age (year)	28.7 ± 4.3	67.8 ± 1.7	< 0.001
Body mass (kg)	61.7 ± 9.8	71.4 ± 11.4	0.6
Body height (cm)	168.0 ± 9.0	170.1 ± 5.7	0.11

### Experimental protocol

**Figure 1:**
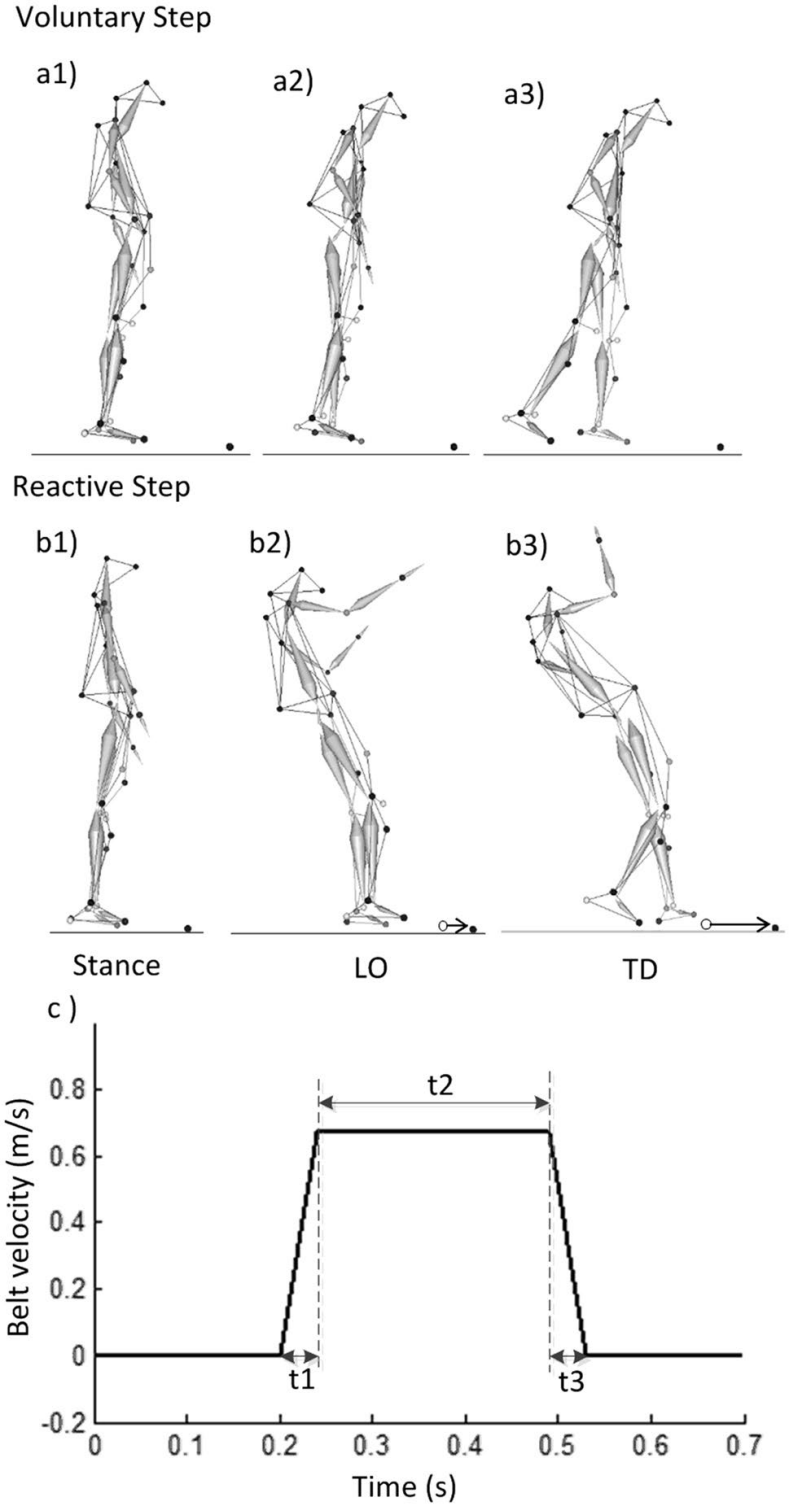
The schematic representation of (**a**) volitional stepping and (**b**) reactive stepping of a representative subject in this study during the time of events of (1) slip onset (for reactive step) or auditory cue onset (for voluntary step), (2) lift-off (LO) of the stepping limb, and (3) touchdown (TD) of the stepping limb. (**c**) The treadmill slip profile for the familiarization trial. The profile contains three phases: t1: acceleration phase (t = 0.04 s, a = 16.75 m/s^2^), t2: constant speed phase (t = 0.25 s, a = 0 m/s^2^), and t3: deceleration phase (t = 0.04 s, a = − 16.75 m/s^2^). The second slip trial contains the same phases but with longer duration and larger acceleration (t1: t = 0.04 s, a = 21.5 m/s^2^; t2: t = 0.33 s, a = 0 m/s^2^; t3: t = 0.04 s, a = − 21.5 m/s^2^).

All participants wore a safety harness and stood on the ACTIVESTEP motorized treadmill (SIMBEX, Lebanon, NH). A baseline trial was first collected for all participants during quiet standing. Next, the participants were asked to take a voluntary step in the backward direction following an auditory cue as rapidly as they could (Fig. 1). The auditory cue (“BEEP” sound) was given within 2–4 s after the start of the trial (indicated by a “START” sound). In this trial, the treadmill belt remained still. There are two voluntary stepping trials in total; participants took a step using their dominant leg in the first trial and then using their subdominant leg in the second trial.

**Figure 2:**
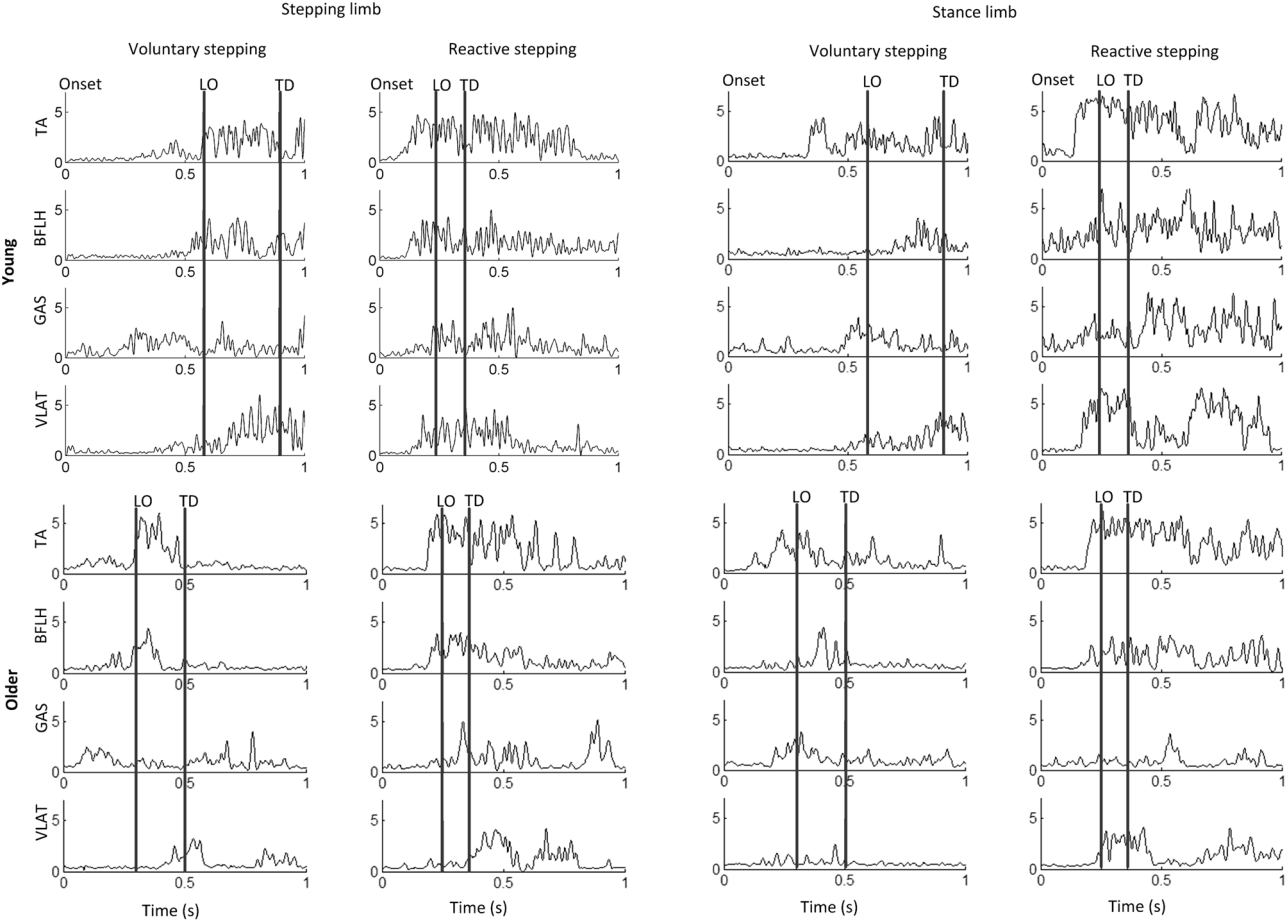
A sample of electromyography (EMG) post-perturbation onset during voluntary and reactive stepping for a young and an older participant. The two vertical bars denote the LO and TD events, respectively. *TA* tibialis anterior, *BFLH* biceps femoris long head, *GAS* medial gastrocnemius, *VLAT* vastus lateralis.

Following the voluntary stepping trials, the participants received two slip-perturbation trials. In these trials, participants were exposed to a sudden, slip-like treadmill perturbation during stance without knowledge on the exact onset time of perturbation. The subjects were told that a slip-like disturbance could occur on any given trial, and that upon experiencing the slip they had to try their best to recover their balance and not fall. The perturbation was triggered at a randomized instant within 2–4 s after the start of each trial. A familiarization trial was given at a lower intensity in order to truly quantify synergies representative of the perturbation recovery response and to minimize the effect of the startle response. The familiarization trial had a belt displacement of 0.19 m, velocity of 0.67 m/s, and acceleration of 16.75 m/s^2^ (Fig. 2). The second perturbation trial was subsequently given at a higher intensity in which the displacement of the belt was 0.38 m, the velocity was 0.86 m/s, and the acceleration was 21.5 m/s^2^. This perturbation trial, after familiarization, was the trial of interest. The intensity for these two kinds of slip trials were chosen following previous locomotor perturbation studies^54,55^. The slip-perturbation occurred when the treadmill belt translated forward resulting in a backward loss of balance, and all participants took a reactive step for fall prevention. The recording duration for both voluntary stepping and reactive stepping trails was 8 s. This study analyzed the second slip-perturbation trial and the voluntary step trial for the same step side as the slip-perturbation trial.

### Data collection and processing

Kinematic data was collected using an eight-camera motion capture system with a sampling rate of 120 Hz (MOTION ANALYSIS corporation, Santa Rosa, CA) and 29 markers from the Helen Hayes marker set were placed on bilateral bony landmarks, the head, and the trunk to compute the joint centers^56^. The raw marker data was low-pass filtered using a fourth order Butterworth filter with a cutoff frequency of 6 Hz^57^, and the kinematic variables were computed using customized MATLAB routines (MATHWORKS, Natick, MA).

DELSYS Trigno surface electromyography (EMG) sensors were used to record EMG activity from four muscles on each leg at 1200 Hz. These muscles were the tibialis anterior (TA), medial gastrocnemius (GAS), vastus lateralis (VLAT), and biceps femoris long head (BFLH). Raw, unrectified EMG signals were first hardware band-pass filtered over a bandwidth of 20–450 Hz, applying a standard mode rejection ratio of > 80 db. After data collection, the EMG data was digital high-pass filtered at 35 Hz and then full-wave rectified. The rectified data was smoothed via a second-order, dual-pass Butterworth low-pass filter with a 40 Hz cutoff frequency^37^. A sample of the filtered EMG signal is demonstrated in Fig. 2.

### Outcome variables

#### Gait-related variables

The crucial time events were foot lift-off (LO) and foot touchdown (TD) after the auditory cue or slip onset, and these events were detected from the Z coordinate of the heel and fifth metatarsal markers. LO was identified when the stepping foot (heel or metatarsal) exceeded more than two standard deviations from their mean baseline value during quiet stance, and TD was identified as the instant of initial contact of the foot with the treadmill when the foot reached the baseline. These event times were manually verified using recorded video. The reaction period was defined as the duration from onset of perturbation or auditory cue (PON) to LO, and the execution period was defined as the duration between LO and TD. Step length was calculated as the heel distance at TD in the anteroposterior (AP) direction. The foot angle was calculated as the angle between the foot segment and the ground in the sagittal plane at TD, and the knee angle was calculated as the angle between the shank segment and the thigh segment in the sagittal plane at TD. The maximum knee and foot angles were calculated as the peak values between PON and TD.

#### Muscle synergy extraction

EMG data from PON to TD were down-sampled by averaging the data in 15 ms bins, and then EMG data from each trial was concatenated to create matrices that were 8 (number of muscles) × n (number of time bins) in size^58^. This time bin method provides focus on the overall tendency of the EMG signals and improves the efficiency of muscle synergy analysis, which was widely used in previous studies^37,59,60^. The 15 ms time bins were selected in order to have sufficient time resolution and to avoid dealing with point to point changes in the highly variable EMG signals^61^. For each subject, the novel reactive stepping trial and the corresponding voluntary stepping trial were used. Considering the difference in the duration of voluntary stepping and reactive stepping as well as the potential effect of time bin number on extracted muscle synergies, a longer time bin (28 ms) was also used for the voluntary stepping trials to verify whether the length of time bin could affect our conclusion. In this case, the number of time bins for both types of stepping are almost the same. The EMG matrices for each subject were then normalized to the corresponding maximum activation in the two non-perturbation (voluntary stepping for both sides) trials^62^. Although it was proposed that normalizing EMG from maximal voluntary isometric contraction (MVIC) was the only valid method for muscle activation level comparison between tasks and individuals^63^, the structure of muscle synergies was reported to be highly similar between this MVIC method and the method used in this study^64^. For normalization, each row (represents a muscle vector for one subject) was divided by this maximum activation for both voluntary and reactive trials. Next, each row was scaled to have unit variance (SD = 1) to ensure that each muscle was equally weighted in the extraction. This unit variance was removed by multiplying their original SD value for each row after muscle synergy extraction, thus changing the muscle synergies back to their original scaling.

Previous studies have shown a fast adaptation to the slip perturbations within 1–3 trials, so in order to avoid a learning effect that could induce modifications in neuromuscular control^65^, only the first perturbation trial (after familiarization trial) was used for muscle synergy analysis. Muscle synergies were extracted from these EMG data matrices for each trial by non-negative matrix factorization (NNMF) using customized Matlab routines^66^, NNMF was a decomposition algorithm used extensively in muscle synergy analysis^58,62,67^. This algorithm assumes that a muscle activation pattern, M, which was evoked by a perturbation (slip or auditory cue) in a given time period is comprised of a linear combination of a few muscle synergies, *w*_*i*_, which are each recruited by a synergy recruitment coefficient, *c*_*i*_. Therefore, a particular muscle activation pattern would be characterized by the following equation: M = *c*_*1*_* w*_*1*_ + *c*_*2*_* w*_*2*_ + ⋯  + *c*_*n*_* w*_*n*_ + *n*.

In this equation, *n* is a scalar of EMG noise, *w*_*i*_ is a vector corresponding to the *i*th muscle synergy, and each *w*_*i*_ was multiplied by a scalar recruitment coefficient, *c*_*i*_. It was determined that the spatial components were considered a fixed time-invariant pattern while the temporal activation coefficients vary across time^33^. *w*_*i*_ was further normalized by its maximal value to guarantee all the values in the structure vector are between 0 and 1. If the average contribution of one component in a muscle synergy was above the threshold of 0.4, it was considered as a major contributor to that synergy^68^.

The number of muscle synergies required to explain any of these datasets was determined by selecting the smallest number of synergies that could adequately reconstruct the muscle responses, which was quantified by the variance accounted for (VAF). To ensure consistency in selecting the number of muscle synergies within each condition, the number of muscle synergies selected was the minimum number at which the muscle synergies accounted for greater than 75% of the VAF in each muscle and exceeded 90% of the overall VAF^37^. As only eight muscles were analyzed in our study, to avoid the underestimation of the muscle synergy number, an individual VAF of 80% and the overall VAF of 90% was used to validate our results.

To facilitate the comparison of muscle coordination patterns between different stepping responses and between different age groups, muscle synergies extracted from voluntary step trials and reactive step trials were first pooled across subjects and then grouped with a hierarchical cluster analysis^58^. The number of clusters for each condition was determined by identifying the minimum number of clusters that partitioned the muscle synergies such that no cluster contained more than one muscle synergy from the same subject. The clustered muscle synergies were ordered by their recruited times. These repertories of muscle synergies were compared between conditions or between age groups.

### Cross validation of the clustered muscle synergies

To verify the robustness of the clustered muscle synergies, we used a cross-validation procedure^35,69^. First, we extracted muscle synergies from a random sub-maximum (80%) dataset for both young and older groups separately, and then we compared these clustered muscle synergies with those exacted from the whole dataset for each condition.

### Statistical analysis

To examine the demographics between young and older participants, their body weight and body height were compared using independent *t* tests.

To investigate the kinematic differences between voluntary stepping and reactive stepping, two-way ANOVA was conducted to compare the age and task effects on the step length, reaction period, execution period, bilateral joint angles (knee and foot) at TD, maximum knee flexion from PON to TD, and maximum plantar flexion from PON to TD. Significant main effects and interactions were resolved using paired and independent *t* tests for within task and between groups, respectively. Benjamini and Yekutieli (B–Y) corrections were applied to adjust the false discovery rate for these Post hoc tests (corrected α = 0.024)^70^. To verify whether there is any difference in performance between right vs. left side stepping for the reactive stepping trials, an independent *t* test was used to compare the above variables between these two different steps for the young and older participants.

To test the differences in muscle synergies between voluntary stepping and reactive stepping as well as between young and older participants, the number of muscle synergies were first compared using a paired Wilcoxon signed rank test between different step types and using Kruskal–Wallis test between age groups. Cohen’s d mean difference effect sizes and power sizes were also calculated to verify the validity of these results. Next, the correlation coefficient (r) was calculated between the average structure vectors (*w*_*i*_) of muscle synergy from the clusters to compare the coordination pattern of muscle synergies. A pair of muscle synergies from different step types or from different age groups were considered similar if they had r > 0.834, which represents statistically significant similarity (p < 0.01)^71^. The effect of muscle synergies on kinematics were also analyzed by comparing each kinematic variable between the subjects with and without using specific muscle synergy.

To verify the results of synergy comparison between step types (based on clustered synergies), the individual muscle synergies from the same subject within each group were also compared by calculating the correlation coefficient (r value) between individual syneriges recuirted in different step types. Due to the individual variability in neuromuscular control, there might be inconsistencies between the individual synergies and the common synergies in each cluster. Therefore, the similarity (r value) between individual muscle synergy and its corresponding clustered muscle synergies were calculated to validate our findings based on common synergies.

Additionally, the extracted muscle synergies using different time bins were also compared to examine the effect of time bins on muscle synergy. For the pairs of similar muscle synergies between age groups and between step types, the peak values, and the corresponding instants of the corresponding recruitment coefficients (*c*_*i*_) for each participant were also compared using independent *t* tests.

## Results

Half of the 20 participants stepped with their right limb in the reactive stepping trials, and others stepped with their left limb. The main effects of age and task as well as age × task interaction is presented in Table 2 for each variable of interest. Regarding the kinematic response during reactive and voluntary stepping in both young and older adults, it was possible to observe that during reactive backward stepping the step length, reaction time, and execution time were shorter compared to during voluntary backward stepping for both young (p < 0.001) and older (p < 0.05) adults. For all the variables mentioned above, no significant difference was found between left and right stepping sides for all the variables analyzed (p > 0.05) following the slip-perturbation for both young and older participants. Conversely, both young and older adults showed a larger knee flexion (p < 0.01 for knee flexion at TD; p < 0.05 for max knee flexion) during reactive stepping compared to the knee flexion observed during voluntary stepping; however, no significant differences in plantar flexion angle were observed between reactive and voluntary stepping (p > 0.05).

**Table 2:** 2-way ANOVA results of all the kinematics and step times. Here SL denotes step length, KF denotes knee flexion, and PF denotes plantarflexion.

Variables	Yong		Older		2-way ANOVA		
	Voluntary	Reactive	Voluntary	Reactive	Age	Task	Interaction
Reaction (ms)	489.2 ± 93.7	210.8 ± 41.4	516.7 ± 86.5	267.6 ± 59.9	0.09	< 0.001	0.52
Execution (ms)	272.5 ± 59.2	155.0 ± 24.2	214.1 ± 63.5	130.8 ± 53.3	0.09	< 0.001	0.20
SL (mm)	457.7 ± 59.7	263.5 ± 110.4	296.4 ± 116.6	162.9 ± 105.5	< 0.001	0.004	0.27
KF at TD (deg)	167.0 ± 13.0	140.7 ± 4.1	163.9 ± 12.8	139.2 ± 18.9	0.53	< 0.001	0.43
Max. KF (deg)	147.2 ± 12.9	120.0 ± 9.8	153.5 ± 16.9	129.2 ± 24.0	0.18	0.012	0.90
PF at TD (deg)	13.6 ± 10.9	11.5 ± 7.3	11.8 ± 8.6	9.4 ± 5.8	0.86	0.36	0.80
Max. PF (deg)	26. 1 ± 7.8	22.3 ± 5.3	23.2 ± 7.6	18.7 ± 5.2	0.15	0.06	0.91

**Figure 3:**
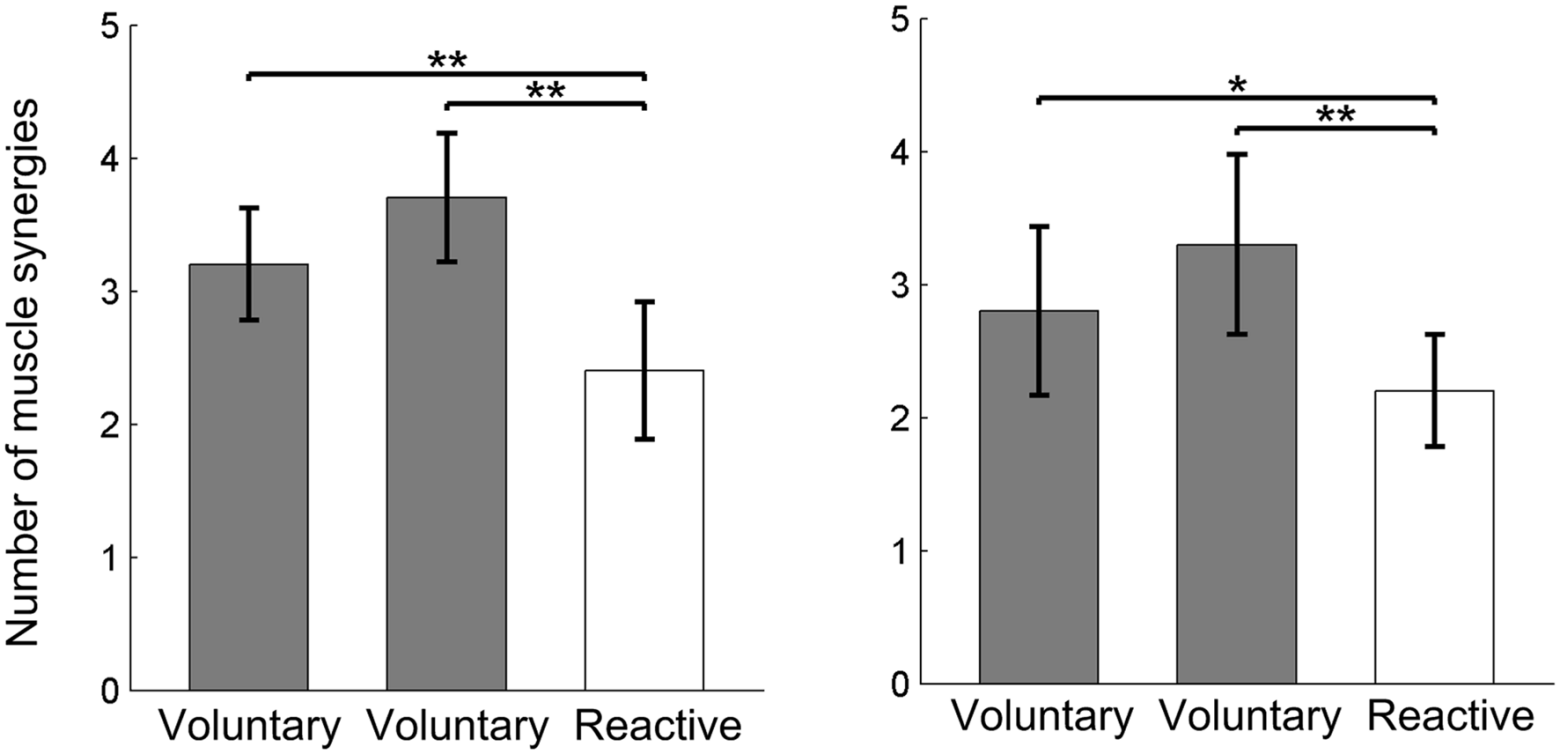
Number of muscle synergies during voluntary (two gray bars with time bin of 28 ms and 15 ms) and reactive (white bar) stepping for each participant (overall VAF > 90%). The left panel shows the comparison of muscle synergy quantities for young participants and the right panel shows the comparison of muscle synergy quantities for older adults. No difference was found between young and older adults for both voluntary and reactive stepping. Black circles denote the number of muscle synergies per trial; **denotes 0.01 > p ≥ 0.001. For the comparison of muscle synergies in older adults: Cohen’s d = 0.87 and 1.92 and Power = 86% and 99%, respectively; for the comparison of muscle synergies young adults: Cohen’s d = 1.02 and 1.58; Power = 90% and 98%, respectively.

**Figure 4:**
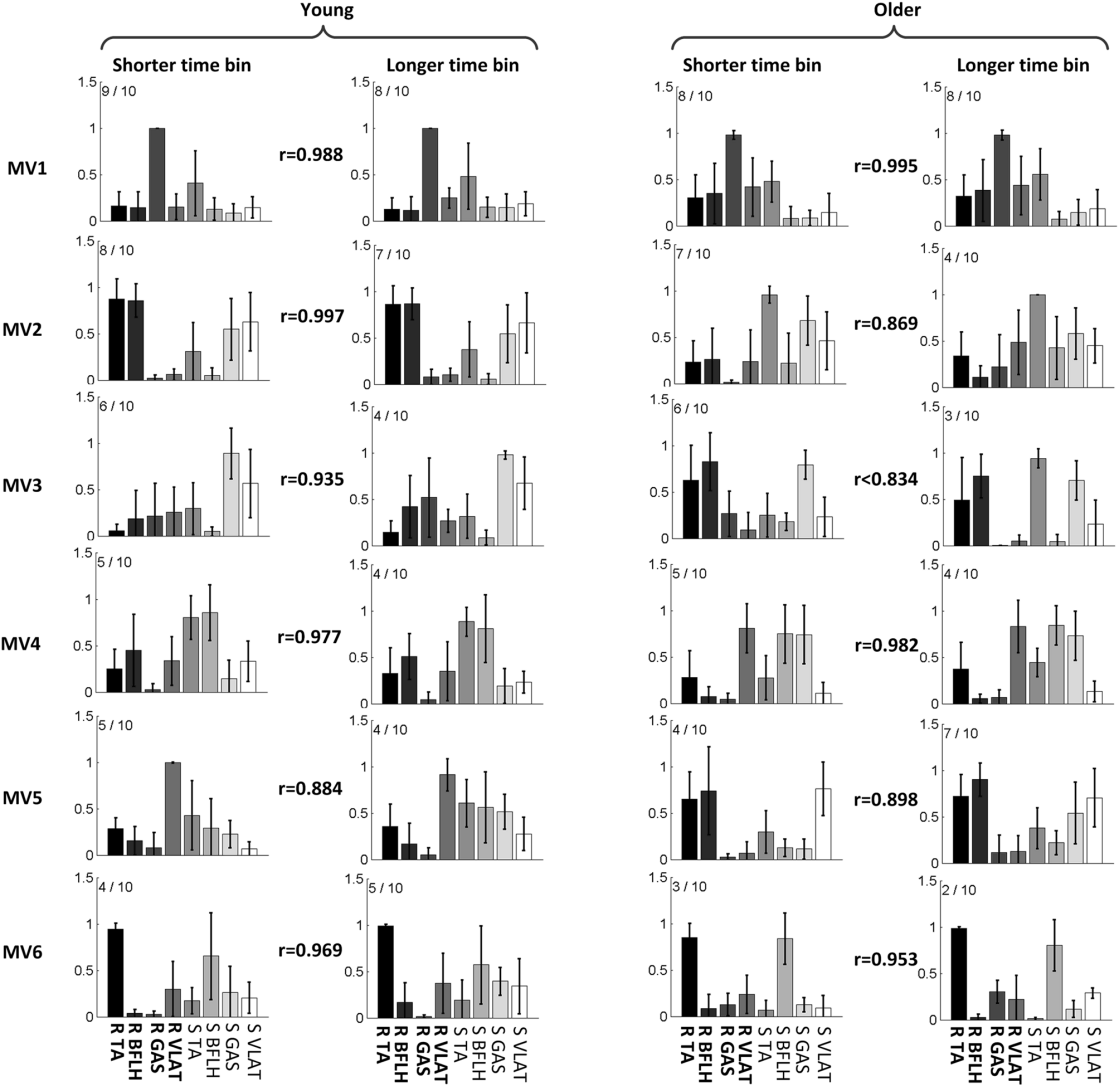
The averaged muscle synergy comparison between voluntary stepping with different time bins for young and older subjects. The muscle synergies based on longer time bin (28 ms) were ordered by their recruited times, x/10 adjacent to each muscle synergy indicate x out of 10 participants recruited that muscle synergy. MV denotes muscle synergy during voluntary stepping. The muscle synergies based on shorter time bin (15 ms) were ordered according to the similarity between them and the ones based on longer time bin. Mean values are represented by the bars in the motor module figure. SDs are represented by the error bars in the motor module figure. The first 4 muscles in each muscle synergy are for the recovery limb (R) and the other 4 are for the stance limb (S).

During voluntary backward stepping, participants used more muscle synergies compared to reactive backward stepping, and these results were observed in both the young (p < 0.01, Cohen’s d > 0.82, and Power > 0.85 for both time bins) and older adults (p < 0.01, Cohen’s d > 0.82, Power > 0.85 for both time bins) groups, while no difference in the muscle synergy numbers was found between young and older adults (p > 0.05, Fig. 3). Additionally, there was very limited effect of time bins on the muscle synergies, among all 12 pairs of muscle synergies for both young and older adults (6 pairs for each group), only 1 out of 12 pairs were not similar (r < 0.834 but > 0.8, Fig. 4) between the time bin of 15 ms and 28 ms, thus, only the muscle synergy based on the time bin of 15 ms was analyzed.

**Figure 5:**
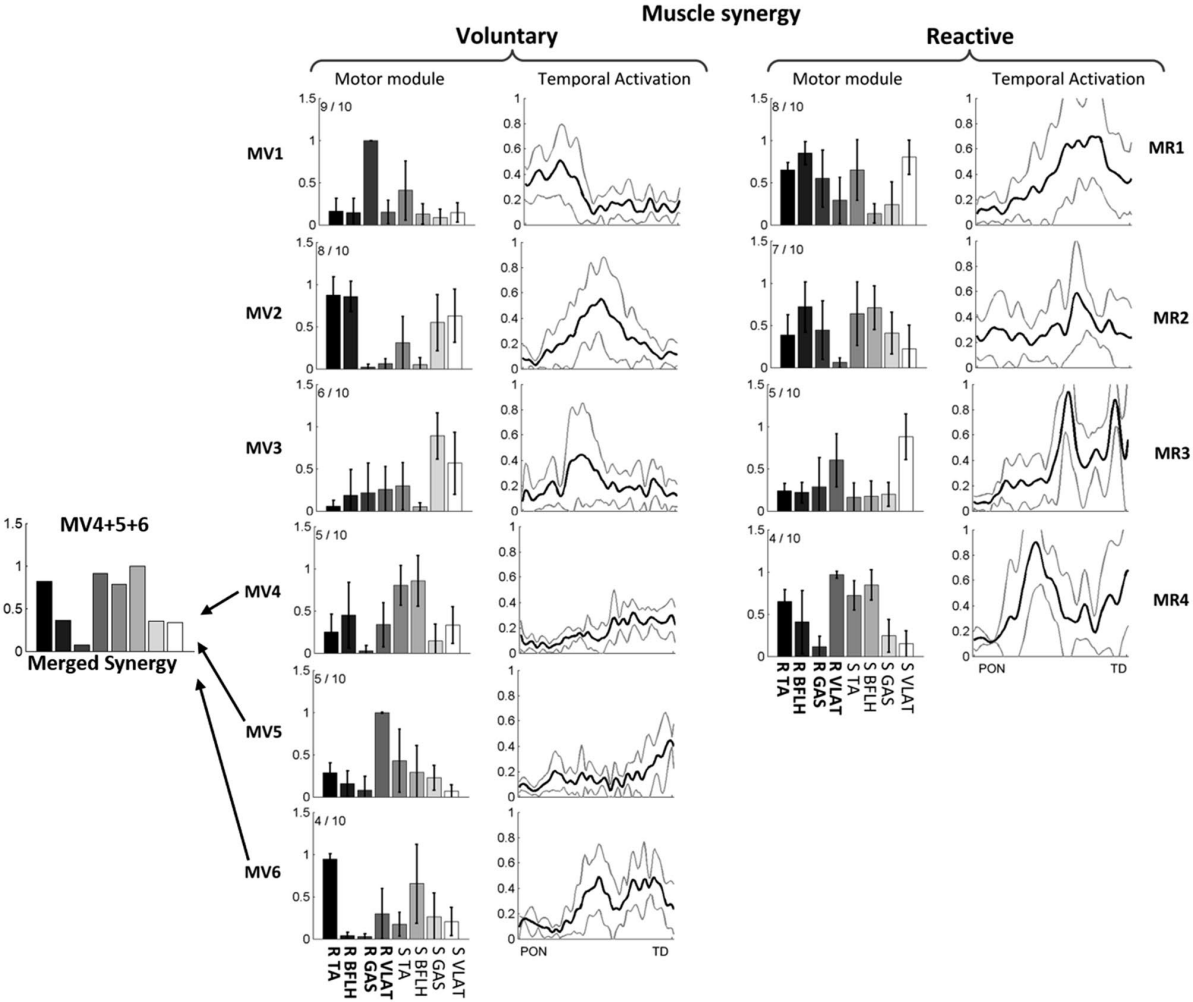
The averaged muscle synergy comparison between voluntary and reactive stepping for young subjects, as well as the cluster analysis of the identified repertories of 6 muscle synergies from the voluntary stepping trials and 5 muscle synergies from the reactive step trials. The muscle synergies were ordered by their recruited times, x/10 adjacent to each muscle synergy indicate x out of 10 participants recruited that muscle synergy. MV denotes muscle synergy during voluntary stepping and MR denotes muscle synergy during reactive stepping. None of these muscle synergies had similar spatial structures (r > 0.834) between the MVs and MRs. While merged muscle synergies of MV4, MV5, and MV6 had a similar structure to MR4 (r = 0.952). The merging was conducted by linearly combining the to-be-merged synergies, and only the merged synergy in voluntary stepping showed a similarity with the muscle synergies in reactive stepping were presented here. Mean values are represented by the bars in the motor module figure and by the black lines in the temporal activation figure. SDs are represented by the error bars in the motor module figure and by the gray lines in the temporal activation figure. The first 4 muscles in each muscle synergy are for the recovery limb (R) and the other 4 are for the stance limb (S).

**Figure 6:**
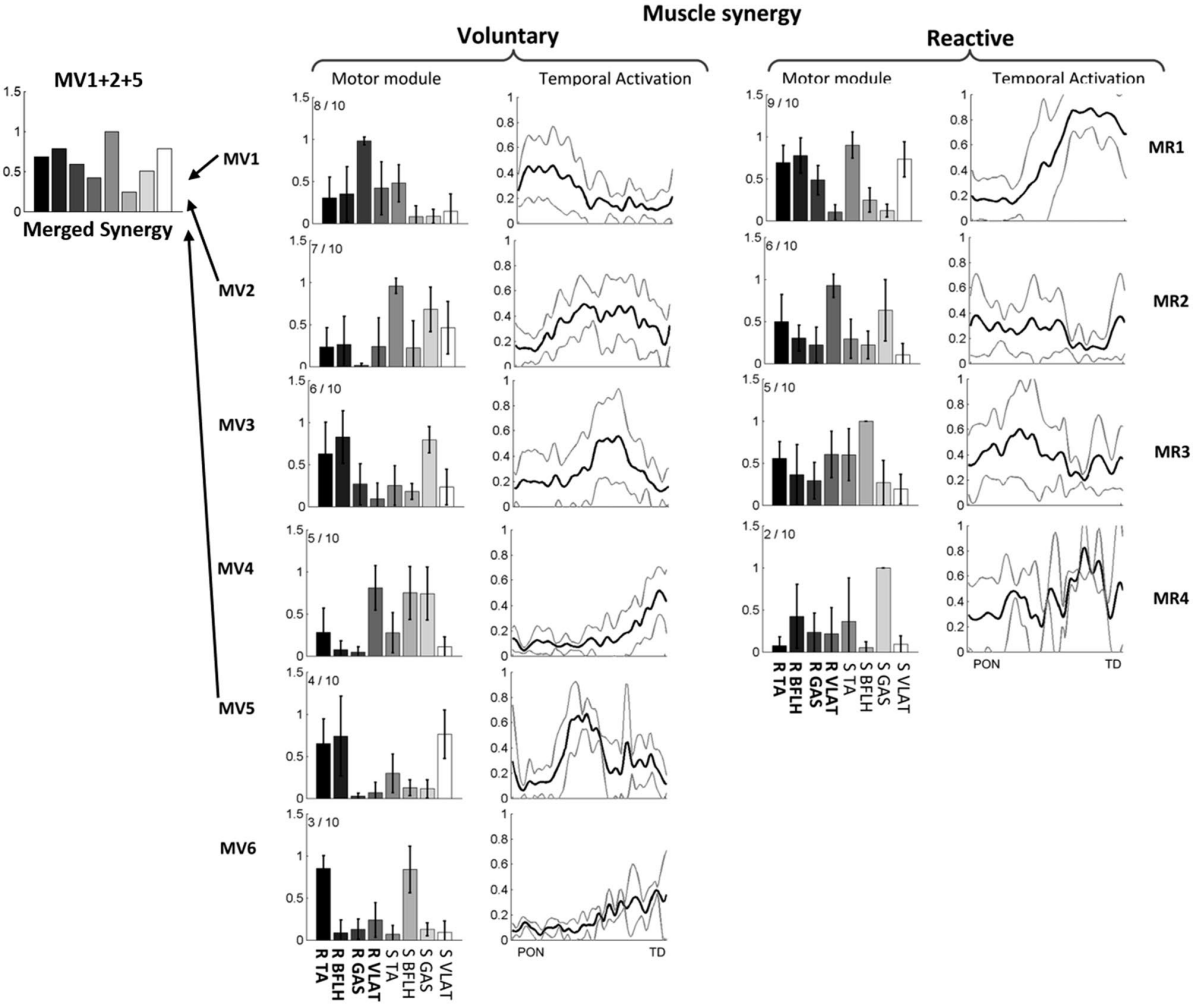
The averaged muscle synergy comparison between voluntary and reactive stepping for older subjects, as well as the cluster analysis of the identified repertories of 6 muscle synergies each from the voluntary and reactive stepping trials. The muscle synergies were ordered by their recruited times, x/10 adjacent to each muscle synergy indicate x out of 10 participants recruited that muscle synergy. MV denotes muscle synergy during voluntary stepping, and MR denotes muscle synergy during reactive stepping. None of these muscle synergies had similar spatial structures (r > 0.834) between the MVs and MRs. While merged muscle synergies of MV1, MV2, and MV5 had a similar structure to MR1 (r = 0.888). Mean values are represented by the bars in the motor module figure and by the black lines in the temporal activation figure. SDs are represented by the error bars in the motor module figure and by the gray lines in the temporal activation figure. The first 4 muscles in each muscle synergy are for the recovery limb (R) and the other 4 are for the stance limb (S).

**Figure 7:**
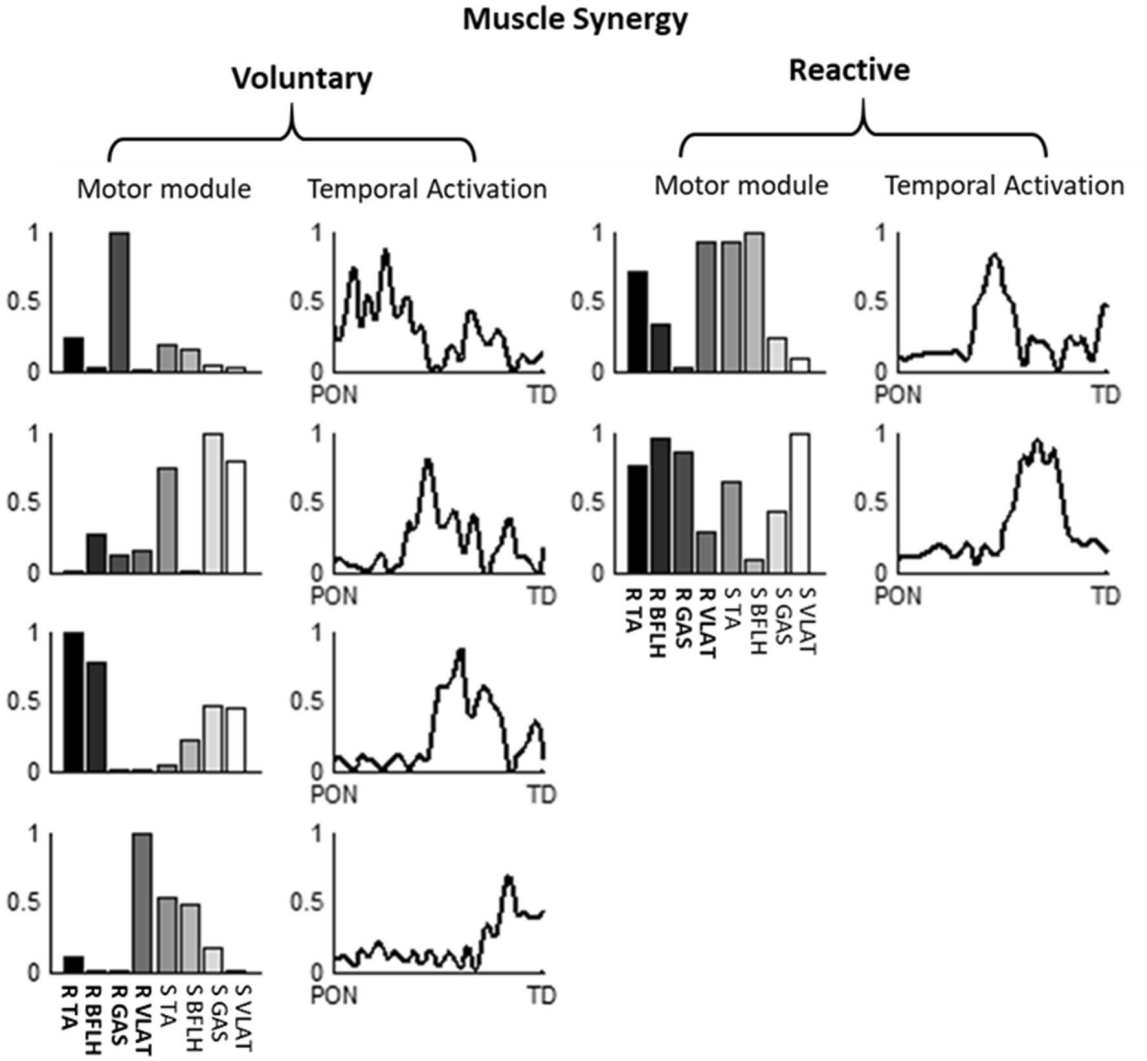
A sample of time organized muscle synergies for voluntary stepping and reactive stepping, respectively. The muscle synergies were ordered by their activation sequence, The first 4 muscles in each muscle synergy are for the recovery limb (R) and the other 4 are for the stance limb (S).

During voluntary backward stepping, none of the grouped muscle synergies recruited by young and older adults were similar to those recruited during reactive stepping (r < 0.834) (Figs. 5 and 6), which was highly consistent with the comparison of individual muscle synergies (Table 1 in Supplementary appendix). Moreover, these correlation coefficients were also consistent with muscle synergies based on a higher VAF (Fig. 1 and Table 2 in the Supplementary appendix). Regarding the muscle activation patterns during voluntary stepping, participants tended to activate fewer muscles than during reactive stepping. Among young participants, 3 out of 4 muscle synergies co-activated more than 5 muscles during reactive stepping (the load > 0.4, shown as MR1, MR2, and MR4 in Fig. 5), but all the synergies co-activated less than 5 muscles during voluntary stepping (MV1-6 in Fig. 5). Similarly, the older participants co-activated 5 muscles in MV1 and MV3 during reactive stepping but not during voluntary stepping (Fig. 6). This co-activation difference could also be found in the individual (not grouped) muscle synergies for a given subject (Fig. 7).

For voluntary stepping, the predominant muscle synergy (MV1, mainly consisted of stepping GAS and stance TA muscle activations) recruited by 90% of young adults (Fig. 5) was similar to the predominant muscle synergy (MV1, mainly consisted of stepping GAS, VLAT, and stance TA muscle activations) recruited by 80% of older participants (Fig. 5) (r = 0.926, Table 3). In addition to this predominant muscle synergy, MV2 (mainly consisted of stepping TA, stepping BFLH, stance GAS, and stance VLAT muscle activations, Fig. 5) recruited by 80% of young participants was similar to MV5 recruited by 40% of older participants (r = 0.858). MV6 (mainly consisted of stepping TA and stance BFLH) recruited by 40% of young participants was similar to MV6 recruited by 30% of older participants (r = 0.936). Further, the most common muscle synergy recruited during reactive stepping was also similar between young (MR1 recruited by 80% of participants, consisted of stepping TA, stepping BFLH, stepping GAS, stance TA, and stance VLAT, Fig. 5) and older (MR1 recruited by 90% of participants, Fig. 6) participants (r = 0.894). Additionally, MR4 (mainly consisted of stepping TA, stepping BFLH, stepping VLAT, stance TA, and stance VLAT) recruited by 40% of young participants was similar to MR3 recruited by 50% of older participants. Among all these synergies, it was found the recruitment of MV4 (consisted of stance BFLH, stance TA and stepping BFLH, Fig. 5) was related to the increase of the execution duration, the recovery step length, and the plantar flexion of the stepping foot (Table 4).

**Table 3:** Similarity between average muscle synergies in young and older participants for both voluntary and reactive stepping trials. The correlation coefficient (r) was calculated between each muscle synergy in young group with all the muscle synergies in older group for voluntary stepping and reactive stepping, respectively. Only the pairs with similarity > 0.834 are shown here.

Step type	Voluntary (15 ms)			Voluntary (28 ms)			Reactive		
Groups	Young	Older	r value	Young	Older	r value	Young	Older	r value
Similarity	MV1	MV1	0.926	MV1	MV1	0.916	MR1	MR1	0.894
	MV2	MV5	0.858	MV2	MV5	0.975	MR4	MR3	0.851
	MV6	MV6	0.936	MV5	MV4	0.869			
				MV6	MV6	0.836

**Table 4:** Comparison of all the kinematics and step times between the young subjects with and without using MV4. Here SL denotes step length, KF denotes knee flexion, and PF denotes plantarflexion.

Voluntary stepping			
variables	Mean ± SD with MV4	Mean ± SD without MV4	P value
SL (mm)	495.2 ± 35.1	420.2 ± 57.3	0.037
Execution (ms)	310 ± 43.5	241.7 ± 43.7	0.038
PF at TD (deg)	22.9 ± 4.3	2.4 ± 3.6	< 0.001

The cross-validation indicated that the clustered muscle synergies from sub-maximum dataset were highly consistent with those extracted from the entire dataset (r > 0.84 for all, Table 5). Furthermore, there was a moderate to high similarity (0.68 ≤ r ≤ 0.92, mean value in Table 6) between the averaged synergy and the individual synergies for each cluster.

**Table 5:** Similarity between average muscle synergies from sub-maximum dataset (80%) and those from entire dataset. The correlation coefficient (r) was calculated for voluntary stepping and reactive stepping in both young and older adults, respectively.

Groups	Young	r value	Older	r value	Young	r value	Older	r value
Similarity	MV1	0.997	MV1	0.994	MR1	0.994	MR1	0.996
	MV2	0.996	MV2	0.963	MR2	0.979	MR2	0.988
	MV3	0.997	MV3	0.950	MR3	0.999	MR3	0.980
	MV4	0.999	MV4	0.999	MR4	0.999	MR4	0.999
	MV5	0.980	MV5	0.969				
	MV6	0.999	MV6	0.891

**Table 6:** Average similarity between the clustered muscle synergy and individual muscle synergies that belong to that cluster. The correlation coefficient (r) was calculated for each of the clustered muscle synergies in both young and older adults. S1-S10 indicates the first to the 10^th^ subjects for both age groups.

Groups	Clustered synergy	Similarity (r) between individual and clustered synergy										Mean ± SD
		S1	S2	S3	S4	S5	S6	S7	S8	S9	S10	
Older	MV1		0.92	0.90	0.84	0.56	0.84	0.75	0.88		0.91	0.82 ± 0.12
	MV2	0.51	0.84	0.74			0.77	0.96	0.87	0.83		0.79 ± 0.14
	MV3	0.72	0.88	0.82	0.98					0.34	0.96	0.78 ± 0.24
	MV4					0.78	0.84		0.91	0.81	0.93	0.85 ± 0.07
	MV5				0.59	0.97		0.93		0.86		0.84 ± 0.17
	MV6			0.94	0.88			0.94				0.92 ± 0.03
	MR1	0.90	0.97	0.82	0.91	0.77	0.95	0.85		0.95	0.91	0.89 ± 0.07
	MR2		0.77	0.79			0.82	0.78		0.88	0.79	0.8 ± 0.04
	MR3	0.39			0.83	0.91		0.83	0.83			0.76 ± 0.21
	MR4								0.91	0.90		0.91 ± 0.002
Young	MV1	0.82	0.94	0.89	0.91	0.77	0.95	0.87	0.87		0.92	0.88 ± 0.06
	MV2	0.88	0.86			0.86	0.92	0.93	0.91	0.85	0.77	0.87 ± 0.05
	MV3	0.95		0.75	0.75		0.89		0.48	0.70		0.75 ± 0.16
	MV4				0.66	0.66		0.78		0.93	0.94	0.79 ± 0.14
	MV5	0.81		0.78			0.94	0.95			0.90	0.88 ± 0.08
	MV6		0.84	0.96	0.71				0.92			0.86 ± 0.11
	MR1	0.71		0.68	0.70	0.60	0.94		0.82	0.84	0.86	0.77 ± 0.11
	MR2	0.57	0.70	0.67		0.33		0.73	0.78		0.97	0.68 ± 0.2
	MR3		0.73	0.97		0.80			0.93	0.47		0.78 ± 0.2
	MR4		0.86		0.90		0.97			0.79		0.88 ± 0.08

## Discussion

This study investigated neuromuscular activity during voluntary and reactive backward stepping in healthy young adults and healthy older adults. Consistent with our first postulation, the results showed that the neuromuscular control during reactive and voluntary stepping response was different for both young and older adults, showing that fewer muscle synergies during reactive stepping compared to voluntary stepping were recruited for both groups. Our second hypothesis was supported in that about half of muscle synergies were different between young and older adults for both voluntary and reactive stepping responses.

### Voluntary stepping vs. reactive stepping

Our results indicated that different muscle synergies were recruited for reactive stepping vs. voluntary stepping in the backward direction, especially the muscle synergies related to knee joint movements. For young participants, the predominant muscle synergy that contributed to knee flexion of the stepping limb during voluntary and reactive stepping was MV2 and MR1, respectively (Fig. 5). Similar synergy activation patterns were observed for the synergies controlling knee flexion in the older adult group and additionally the magnitude of reactive stepping synergy MV3 was lower than the voluntary stepping synergy MR1 (Fig. 6). Thus, the larger knee flexion observed in both young and older adults might be attributed to changes in neuromuscular control. As voluntary stepping contains two phases, a knee flexion in the early swing phase and a knee extension in the late swing phase. One might not have sufficient time to extend their knee joint during reactive stepping due to the rapid need to establish double support to regain stability and enhance limb support. This is consistent with the finding in this investigation and prior studies demonstrating that reactive backward stepping has decreased preparation and execution times compared to voluntary stepping^20,72^. In addition, it is known that terminal knee extension is crucial for increasing step length^73^, thus, the increased knee flexion in the reactive step and lack of terminal knee extension could explain the shortened step length of the reactive step compared to the volitional step.

Furthermore, another difference between the two types of stepping was the possible less complex neuromuscular control (fewer muscle synergies; Fig. 3) but higher levels of muscle co-activation (MR1-2 for young adults; MR1, MR4 for older adults) that was observed for the reactive stepping compared to voluntary stepping. It has been previously described that an increase in muscle co-activation, especially the co-activation of antagonist muscles, was a reactive mechanism to increase joint stiffness^49^. As short-range stiffness can provide stability against external perturbation during the neuromechanically response delay^74^, the muscle co-activation could enhance the joint stability. This co-activation strategy might not be an optimal solution for fall prevention, as higher level of muscle co-activation might be a sign of lower adaptability (e.g., the ability of the CNS to rapidly change sensorimotor responses to impending environmental constraint)^75^. However, without prior knowledge of upcoming perturbation or environmental constraint, it is difficult for the subjects to adopt an optimal recovery strategy (longer step length with less co-activation) when they encounter an unexpected perturbation with higher intensity. Hence, in this case, the short-range co-activation in the perturbed limbs might be a first line of defense strategy to enhance limb support, regain stability, and prevent falling.

Previous studies have postulated that a common set of muscle synergies can be recruited for voluntary and reactive motor behaviors^37,67^, which was inconsistent with our findings that different muscle synergies were recruited for voluntary and reactive stepping. One possible reason for this inconsistent could be that the perturbation intensity (displacement = 0.124 m; velocity = 0.4 m/s; acceleration = 6.86 m/s^2^) used in previous studies^37,67^ was lower than the intensity used in our protocol (displacement = 0.38 m; velocity = 0.86 m/s; acceleration = 21.5 m/s^2^). It was reported that a small perturbation with the displacement of 0.1 m and the velocity of 0.4 m/s was not able to trigger a recovery step. As individuals could successfully recover their balance by rotating at the ankle joint with legs straight like an inverted pendulum (ankle strategy), or thrusting the pelvis forward and bend the knees (hip strategy) to shift the center of mass (COM) forward and within the displacing/displaced base of support. Thus, the step (“unnecessary stepping”)^76^ following small perturbation might be mainly for posture adjustment or induced by fear of fall. However, a protective step is necessary to regain stability following a perturbations with higher intensity inducing a balance loss (e.g., COM state travelling outside the base of support)^77^. The stepping strategy, recovery step length, trunk angle at recovery touchdown, and lower limb joint moments and powers are all reported to increase with perturbation intensity^78,79^. Additionally, a previous study found that with the increase in perturbation intensity there were marked changes in peak power and latency of the N1 potential (a cortical Evoque-related potential) in the electroencephalogram, which indicated that even the cortical responses undergo modulation with change in the perturbation intensity^80^. Therefore, it is reasonable to postulate that the muscle synergies during reactive stepping might change with an increase in perturbation intensity. While there is another possibility that the perturbation might not change the structure of muscle synergies but rather merge parts of the muscle synergies in the voluntary stepping trials. For the young adults, the linear combination of MV4, MV5, and MV6 were similar to MR4 (r = 0952), and the linear combination of MV1, MV2, and MV5 were similar to MR1 for the older adults (r = 0888). These results were consistent with the study by Cheung et al., in which the authors stated that merging of muscle synergies may be a mechanism to accommodate the change of limb biomechanics^40^.

### Aging effects on backward stepping

Consistent with previous findings that have shown that balance strategies during gait vary according to age^81^, our results also showed differences in parts (≤ 50%) of the muscle synergy structures between young and older participants, while the coordination of predominant muscle synergies was similar between age groups for both voluntary stepping and reactive stepping (Figs. 5 and 6). Contrary to the task differences (voluntary vs. reactive stepping) seen for muscle synergies, our results indicate aging leads to less changes in the structure of muscle synergies. Considering MV3 in young adults and MV2 in older adults for example (r < 0.834 between them), the only difference between these two synergies was that the weight of muscle TA in the stance limb was larger in older adults than young adults. Similarly, the MV4 in older adults showed a larger weight of muscle BFLH and GAS in the stance limb than the MV5 in young adults. The changes in parts of the muscle synergies might be attributed to age-related muscle function impairments caused by muscle atrophy and degeneration in contraction velocity^82^. These changes in muscle synergies might lead to the difference in step length between young and older adults. No similarity was found between voluntary and reactive stepping for the effect of tasks on muscle synergies; however, half of muscle synergies were found to be similar between aging groups. According to these results, it is possible to indicate that aging has lesser effect on the muscle synergies compared to tasks.

Both kinematic variables and clustered muscle synergies showed a relatively larger within-group variability, especially the step length during voluntary stepping. Our results indicated that the co-activation of stance BFLH, stance TA and step BFLH muscles (MV4 in Fig. 5) might help to increase the execution duration and enlarge the step length. While only 5 out of 10 young subjects recruited this synergy, which would increase the variability in the kinematic variables. Interestingly, no older subjects recruited such a muscle synergy during their voluntary stepping, indicating that the deterioration in stepping performance might be induced by the failure in recruitment of proper muscle synergies (e.g., MV4 in Fig. 5). The variability in muscle synergies was mainly related to few subjects whose synergies have a lower similarity with the cluttered one (e.g., S1 for older and S5 for young subjects in Table 6). With individual kinematic data analysis, it was found these two subjects showed a different performance compared to others. For example, the young subject (S5) showed the smallest knee angle in both voluntary stepping and reactive stepping, and the older subject (S1) showed the smallest knee angle and longest reaction time in voluntary and showed a smaller knee angle in the reactive stepping. These inter-individual differences might be induced by the motor abundance, which lead to the larger variability in the clustered muscle synergies. However, most of the extracted individual muscle synergies still showed a moderate to high similarity to their clustered synergies, indicating that the larger within-group variability would have limited effects on the conclusion.

There are several limitations in this study. First, only 10 subjects for each group were included in this study. Although cross-validation showed that the exacted muscle synergies were robust, the small sample size might still affect the validation of our conclusion, therefore, future research with a larger sample size is necessary to confirm these results. Second, the EMG matrices were normalized from the maximal activation during voluntary stepping trials but not from maximal voluntary isometric contraction (MVIC), which could affect the relative weightings of muscle synergies as the maximal activation during voluntary movements could not be always proportional to the MVIC. Although the structure of muscle synergies was reported to be highly similar between these two methods^63^, future study based on MVIC method need to be conducted to verify our conclusion. Third, the muscle synergy was only extracted using a single trial. A prior study has suggested that repeated trials or extended periods of time was needed to capture the inherent variability of motor commend signals, which is important for estimating the muscle synergies^83^. However, it is known that there is a significant change in perturbation-induced responses after exposure to a very first novel trial with same intensity, known as the first-trial effect^65,84,85^. In this study, to avoid the effect of repeated trials on the muscle synergies, only data from the novel perturbation trial was used and the same was done for voluntary stepping to maintain similarity in analysis. Further, it should be noted that the dimensionality of extracted muscle synergies might not be influenced by the number of trials or step cycles used for EMG factorization (similarity > 0.85 between single and concatenated trials)^86^. Thus, the single trial analysis used in this study should have limited effect on our results. Last, the limited number of muscles recorded could be seen as another limitation of the study, which could lead to an underestimation of the number of muscle synergies recruited during walking and slip responses^87^. However, both the number of muscles and the choice of the muscles could impact the results of the muscle synergies. The muscles included in the present analysis can be considered to be among the dominant muscles involved in the slipping response^58^, which may help offset the effect of a limited number of muscles being used in the muscle synergy analysis. Furthermore, a higher individual VAF (80%) was used to verify the exacted muscle synergies, the results based on the new VAF was highly consistent with our finding (Fig. 1 and Table 2 in the Supplementary appendix), indicating that the number of muscles has limited effects on our conclusion. To further verify our results, more muscles contributed to postural balance control (e.g., trunk muscles) will be included in future studies.

In conclusion, this study investigated the differences in neuromuscular control between voluntary and reactive backward stepping. Our results indicate that muscle synergies were not spatially fixed for lower limb tasks, especially for a large, unexpected perturbation. In the case of this unexpected perturbation, more muscles co-activate with less complexity during the reactive stepping response than during voluntary stepping have a greater chance to resist instability. Our results also indicate that the coordination of muscle synergies are less affected by age than by task constraints.

## Supplementary Information

Supplementary Information.

## References

[1] Rubenstein LZ (2006). Falls in older people: Epidemiology, risk factors and strategies for prevention. Age Ageing.

[2] Tuunainen E, Rasku J, Jantti P, Pyykko I (2014). Risk factors of falls in community dwelling active elderly. Auris Nasus Larynx.

[3] Robinovitch SN (2013). Video capture of the circumstances of falls in elderly people residing in long-term care: An observational study. Lancet.

[4] Stokes HE, Thompson JD, Franz JR (2017). The neuromuscular origins of kinematic variability during perturbed walking. Sci. Rep..

[5] Rankin BL, Buffo SK, Dean JC (2014). A neuromechanical strategy for mediolateral foot placement in walking humans. J. Neurophysiol..

[6] Maki BE, Edmondstone MA, McIlroy WE (2000). Age-related differences in laterally directed compensatory stepping behavior. J. Gerontol. A-Biol..

[7] Wang S, Varas-Diaz G, Dusane S, Wang Y, Bhatt T (2019). Slip-induced fall-risk assessment based on regular gait pattern in older adults. J. Biomech..

[8] Maki BE (1997). Gait changes in older adults: Predictors of falls or indicators of fear?. J. Am. Geriatr. Soc..

[9] Anderson JG, Gilbert FS (1989). Communication-skills training with alcoholics for improving performance of 2 of the alcoholics anonymous recovery steps. J. Stud. Alcohol.

[10] Chomiak T, Watts A, Meyer N, Pereira FV, Hu B (2017). A training approach to improve stepping automaticity while dual-tasking in Parkinson's disease. Medicine.

[11] Creath RA (2013). Self-triggered assistive stimulus training improves step initiation in persons with Parkinson's disease. J. Neuroeng. Rehabil..

[12] Elbar O (2013). A water-based training program that includes perturbation exercises improves speed of voluntary stepping in older adults: A randomized controlled cross-over trial. Arch. Gerontol. Geriatr..

[13] Park GD, Choi JU, Kim YM (2016). The effects of multidirectional stepping training on balance, gait ability, and falls efficacy following stroke. J. Phys. Ther. Sci..

[14] Pai YC, Bhatt TS (2007). Repeated-slip training: An emerging paradigm for prevention of slip-related falls among older adults. Phys. Ther..

[15] Jobges M (2004). Repetitive training of compensatory steps: A therapeutic approach for postural instability in Parkinson's disease. J. Neurol. Neurosurg. Psychiatry.

[16] Nnodim JO (2006). Dynamic balance and stepping versus tai chi training to improve balance and stepping in at-risk older adults. J. Am. Geriatr. Soc..

[17] Shen X, Mak MKY (2012). Repetitive step training with preparatory signals improves stability limits in patients with Parkinson's disease. J. Rehabil. Med..

[18] Rogers MW, Johnson ME, Martinez KM, Mille ML, Hedman LD (2003). Step training improves the speed of voluntary step initiation in aging. J. Gerontol. A-Biol..

[19] Okubo Y, Schoene D, Lord SR (2017). Step training improves reaction time, gait and balance and reduces falls in older people: A systematic review and meta-analysis. Br. J. Sport Med..

[20] Tisserand R (2015). Comparison between investigations of induced stepping postural responses and voluntary steps to better detect community-dwelling elderly fallers. Neurophysiol. Clin..

[21] Mille ML, Simoneau M, Rogers MW (2014). Postural dependence of human locomotion during gait initiation. J. Neurophysiol..

[22] Massion J, Alexandrov A, Frolov A (2004). Why and how are posture and movement coordinated?. Prog. Brain Res..

[23] Rogers MW, Hedman LD, Johnson ME, Martinez KM, Mille ML (2003). Triggering of protective stepping for the control of human balance: Age and contextual dependence. Cogn. Brain Res..

[24] Rogers MW, Hedman LD, Johnson ME, Cain TD, Hanke TA (2001). Lateral stability during forward-induced stepping for dynamic balance recovery in young and older adults. J. Gerontol. A-Biol..

[25] McIlroy WE, Maki BE (1999). The control of lateral stability during rapid stepping reactions evoked by antero-posterior perturbation: Does anticipatory control play a role?. Gait Posture.

[26] Barrett G, Shibasaki H, Neshige R (1986). Cortical potentials preceding voluntary movement: Evidence for three periods of preparation in man. Electroencephalogr. Clin. Neurophysiol..

[27] Jacobs JV, Horak FB (2007). Cortical control of postural responses. J. Neural Transm..

[28] Dietz V, Quintern J, Berger W (1984). Cerebral evoked-potentials associated with the compensatory reactions following stance and gait perturbation. Neurosci. Lett..

[29] Dietz V, Quintern J, Berger W, Schenck E (1985). Cerebral potentials and leg muscle Emg responses associated with stance perturbation. Exp. Brain Res..

[30] Vieira MF (2017). Gait stability, variability and complexity on inclined surfaces. J. Biomech..

[31] Safavynia SA, Ting LH (2013). Long-latency muscle activity reflects continuous, delayed sensorimotor feedback of task-level and not joint-level error. J. Neurophysiol..

[32] Safavynia SA, Ting LH (2013). Sensorimotor feedback based on task-relevant error robustly predicts temporal recruitment and multidirectional tuning of muscle synergies. J. Neurophysiol..

[33] Torres-Oviedo G, Ting LH (2007). Muscle synergies characterizing human postural responses. J. Neurophysiol..

[34] Cappellini G, Ivanenko YP, Poppele RE, Lacquaniti F (2006). Motor patterns in human walking and running. J. Neurophysiol..

[35] Cheung VCK, d'Avella A, Tresch MC, Bizzi E (2005). Central and sensory contributions to the activation and organization of muscle synergies during natural motor behaviors. J. Neurosci..

[36] Wang Y, Zatsiorsky VM, Latash ML (2006). Muscle synergies involved in preparation to a step made under the self-paced and reaction time instructions. Clin. Neurophysiol..

[37] Chvatal SA, Ting LH (2013). Common muscle synergies for balance and walking. Front. Comput. Neurosci..

[38] Wang Y, Watanabe K, Asaka T (2016). Muscle synergies underlying control of taking a step during support surface translation. Eur. J. Appl. Physiol..

[39] Crenshaw JR, Grabiner MD (2014). The influence of age on the thresholds of compensatory stepping and dynamic stability maintenance. Gait Posture.

[40] Cheung VC (2020). Plasticity of muscle synergies through fractionation and merging during development and training of human runners. Nat. Commun..

[41] Springer S (2006). Dual-tasking effects on gait variability: The role of aging, falls, and executive function. Mov. Disord..

[42] Era P, Heikkinen E (1985). Postural sway during standing and unexpected disturbance of balance in random samples of men of different ages. J. Gerontol..

[43] Maeda Y (2015). Age-related changes in dynamic postural control ability in the presence of sensory perturbation. J. Med. Biol. Eng..

[44] Bayram MB, Siemionow V, Yue GH (2015). Weakening of corticomuscular signal coupling during voluntary motor action in aging. J. Gerontol. A-Biol..

[45] de Lima-Pardini AC (2014). Aging increases flexibility of postural reactive responses based on constraints imposed by a manual task. Front. Aging Neurosci..

[46] Kwon M, Chen YT, Fox EJ, Christou EA (2014). Aging and limb alter the neuromuscular control of goal-directed movements. Exp. Brain Res..

[47] Tieland M, Trouwborst I, Clark BC (2018). Skeletal muscle performance and ageing. J. Cachexia Sarcopenia Muscle.

[48] Vernooij CA, Rao G, Berton E, Retornaz F, Temprado JJ (2016). The effect of aging on muscular dynamics underlying movement patterns changes. Front. Aging Neurosci..

[49] Hortobagyi T, DeVita P (2000). Muscle pre- and coactivity during downward stepping are associated with leg stiffness in aging. J. Electromyogr. Kinesiol..

[50] Thelen DG (2000). Muscle activities used by young and old adults when stepping to regain balance during a forward fall. J. Electromyogr. Kinesiol..

[51] Allen JL, Kautz SA, Neptune RR (2013). The influence of merged muscle excitation modules on post-stroke hemiparetic walking performance. Clin. Biomech. (Bristol, Avon).

[52] Steele KM, Rozumalski A, Schwartz MH (2015). Muscle synergies and complexity of neuromuscular control during gait in cerebral palsy. Dev. Med. Child Neurol..

[53] Cruz-Montecinos C, Perez-Alenda S, Cerda M, Maas H (2019). Neuromuscular control during gait in people with haemophilic arthropathy. Haemophilia.

[54] Patel PJ, Bhatt T (2015). Attentional demands of perturbation evoked compensatory stepping responses: Examining cognitive-motor interference to large magnitude forward perturbations. J. Motor Behav..

[55] Patel P, Bhatt T (2015). Adaptation to large-magnitude treadmill-based perturbations: Improvements in reactive balance response. Physiol. Rep..

[56] Davis RB, Ounpuu S, Tyburski D, Gage JR (1991). A gait analysis data-collection and reduction technique. Hum. Mov. Sci..

[57] Pai YC, Wening JD, Runtz EF, Iqbal K, Pavol MJ (2003). Role of feedforward control of movement stability in reducing slip-related balance loss and falls among older adults. J. Neurophysiol..

[58] Sawers A, Pai YC, Bhatt T, Ting LH (2017). Neuromuscular responses differ between slip-induced falls and recoveries in older adults. J. Neurophysiol..

[59] Vernooij CA (2016). Functional coordination of muscles underlying changes in behavioural dynamics. Sci. Rep. U.K..

[60] Gritsenko V, Mushahwar V, Prochazka A (2001). Adaptive changes in locomotor control after partial denervation of triceps surae muscles in the cat. J. Physiol. Lond..

[61] Asaka T, Wang Y, Fukushima J, Latash ML (2008). Learning effects on muscle modes and multi-mode postural synergies. Exp. Brain Res..

[62] Clark DJ, Ting LH, Zajac FE, Neptune RR, Kautz SA (2010). Merging of healthy motor modules predicts reduced locomotor performance and muscle coordination complexity post-stroke. J. Neurophysiol..

[63] Halaki M, G. K. Normalization of EMG singals: to normalize or not to normalize and what to normalize to. *Computational intelligence in electromyography analysis-a perspective on current applications and future challenges*, 175–194 (2012).

[64] Kieliba P (2018). How are muscle synergies affected by electromyography pre-processing?. IEEE Trans. Neural Syst. Rehabil. Eng..

[65] Liu X, Reschechtko S, Wang SJ, Pai YC (2017). The recovery response to a novel unannounced laboratory-induced slip: The "first trial effect" in older adults. Clin. Biomech..

[66] Lee DD, Seung HS (1999). Learning the parts of objects by non-negative matrix factorization. Nature.

[67] Chvatal SA, Ting LH (2012). Voluntary and reactive recruitment of locomotor muscle synergies during perturbed walking. J. Neurosci..

[68] Neptune RR, Clark DJ, Kautz SA (2009). Modular control of human walking: A simulation study. J. Biomech..

[69] Hug F, Turpin NA, Couturier A, Dorel S (2011). Consistency of muscle synergies during pedaling across different mechanical constraints. J. Neurophysiol..

[70] Benjamini Y, Yekutieli D (2001). The control of the false discovery rate in multiple testing under dependency. Ann. Stat..

[71] Chvatal SA, Torres-Oviedo G, Safavynia SA, Ting LH (2011). Common muscle synergies for control of center of mass and force in nonstepping and stepping postural behaviors. J. Neurophysiol..

[72] Martinez KM, Mille ML, Zhang YH, Rogers MW (2013). Stepping in persons poststroke: Comparison of voluntary and perturbation-induced responses. Arch. Phys. Med. Rehabil..

[73] Faber IR, Nienhuis B, Rijs NP, Geurts AC, Duysens J (2008). Is the modified Tardieu scale in semi-standing position better associated with knee extension and hamstring activity in terminal swing than the supine Tardieu?. Dev. Med. Child Neurol..

[74] De Groote F, Allen JL, Ting LH (2017). Contribution of muscle short-range stiffness to initial changes in joint kinetics and kinematics during perturbations to standing balance: A simulation study. J. Biomech..

[75] Houdijk H (2012). Assessing gait adaptability in people with a unilateral amputation on an instrumented treadmill with a projected visual context. Phys. Ther..

[76] Song H (2019). Evolutionary balance between LRR domain loss and young NBS-LRR genes production governs disease resistance in *Arachis**hypogaea* cv. Tifrunner. BMC Genomics.

[77] Horak FB (2006). Postural orientation and equilibrium: What do we need to know about neural control of balance to prevent falls?. Age Ageing.

[78] Madigan ML, Lloyd EM (2005). Age-related differences in peak joint torques during the support phase of single-step recovery from a forward fall. J. Gerontol. A-Biol..

[79] Carty CP, Cronin NJ, Lichtwark GA, Mills PM, Barrett RS (2012). Lower limb muscle moments and power during recovery from forward loss of balance in male and female single and multiple steppers. Clin. Biomech..

[80] Solis-Escalante T, Stokkermans M, Cohen MX, Weerdesteyn V (2020). Cortical responses to whole-body balance perturbations index perturbation magnitude and predict reactive stepping behavior. Eur. J. Neurosci..

[81] Shkuratova N, Morris ME, Huxham F (2004). Effects of age on balance control during walking. Arch. Phys. Med. Rehabil..

[82] Krivickas LS (2001). Age- and gender-related differences in maximum shortening velocity of skeletal muscle fibers. Am. J. Phys. Med. Rehabil..

[83] Steele KM, Tresch MC, Perreault EJ (2015). Consequences of biomechanically constrained tasks in the design and interpretation of synergy analyses. J. Neurophysiol..

[84] Battilana F (2020). Exercise-linked improvement in age-associated loss of balance is associated with increased vestibular input to motor neurons. Aging Cell.

[85] Karakoc K, Mujdeci B (2020). Evaluation of balance in children with sensorineural hearing loss according to age. Am. J. Otolaryngol..

[86] Oliveira AS, Gizzi L, Farina D, Kersting UG (2014). Motor modules of human locomotion: Influence of EMG averaging, concatenation, and number of step cycles. Front. Hum. Neurosci..

[87] Steele KM, Tresch MC, Perreault EJ (2013). The number and choice of muscles impact the results of muscle synergy analyses. Front. Comput. Neurosci..

