# Supplementary material for: Muscle synergy differences between voluntary and reactive backward stepping
Source: Sci Rep. 2021 Jul 29;11:15462. doi: 10.1038/s41598-021-94699-z (PMC8322057; doi:10.1038/s41598-021-94699-z)

**Supplementary appendix**

Table 1. Number of pairs with similarity > 0.834 between individual muscle synergies between voluntary and reactive stepping trials for young and older participants.

| Subject | 1 | 2 | 3 | 4 | 5 | 6 | 7 | 8 | 9 | 10 |
| --- | --- | --- | --- | --- | --- | --- | --- | --- | --- | --- |
| Young(27ms) | 0 | 0 | 0 | 0 | 0 | 0 | 0 | 0 | 0 | 0 |
| Older(27ms) | 0 | 0 | 0 | 0 | 0 | 0 | 0 | 0 | 1 | 0 |
| Young(15ms) | 0 | 0 | 0 | 0 | 0 | 0 | 0 | 0 | 0 | 0 |
| Older(15ms) | 0 | 0 | 0 | 0 | 0 | 0 | 0 | 0 | 2 | 0 |

Table 2. Similarity between average muscle synergies based on the individual VAF of 80% in young and older participants for both voluntary and reactive stepping trials. The correlation coefficient (r) was calculated between each muscle synergy in young group with all the muscle synergies in older group for voluntary stepping and reactive stepping, respectively. Only the pairs with similarity > 0.834 are shown here.

| Step type | Voluntary | | | Reactive | | |
| --- | --- | --- | --- | --- | --- | --- |
| Groups | Young | Older | r value | Young | Older | r value |
| Similarity | MV1 | MV1 | 0.924 | MR3 | MR1 | 0.861 |
|  | MV2 | MV5 | 0.873 | MR5 | MR2 | 0.954 |
|  | MV5 | MV6 | 0.926 |  |  |  |

Figure 1. The averaged muscle synergy based on the individual VAF of 80% in young and older participants for both voluntary and reactive stepping trials. The muscle synergies were ordered by their recruited times, x/10 adjacent to each muscle synergy indicate x out of 10 participants recruited that muscle synergy. MV denotes muscle synergy during voluntary stepping and MR denotes muscle synergy during reactive stepping.


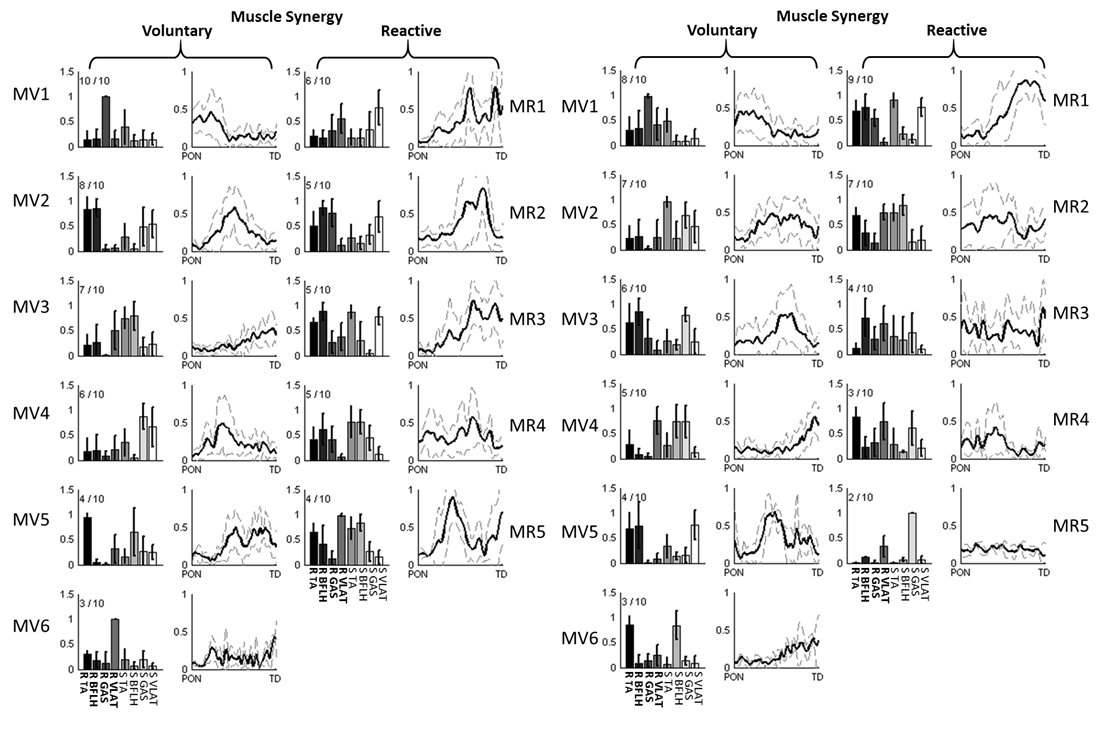

Supplement: Supplementary file 1 — Supplementary Information. [file 41598_2021_94699_MOESM1_ESM.docx]
